# Clinical and Molecular Features of Morpheaform Basal Cell Carcinoma: A Systematic Review

**DOI:** 10.3390/curroncol30110720

**Published:** 2023-11-13

**Authors:** Santina Conte, Sarah Ghezelbash, Bonika Nallanathan, Philippe Lefrançois

**Affiliations:** 1 Faculty of Medicine and Health Sciences, McGill University, Montreal, QC H3G 2M1, Canada; santina.conte@mail.mcgill.ca; 2 Cancer Axis, Lady Davis Institute for Medical Research, Montreal, QC H3T 1E2, Canada; sarah.ghezelbash@mail.mcgill.ca (S.G.); bonika.nallanathan@mail.mcgill.ca (B.N.); 3 Division of Experimental Medicine, Department of Medicine, McGill University, Montreal, QC H3G 2M1, Canada; 4 Division of Dermatology, Department of Medicine, McGill University, Montreal, QC H3G 2M1, Canada; 5 Division of Dermatology, Department of Medicine, Jewish General Hospital, Montreal, QC H3T 1E2, Canada

**Keywords:** morpheaform basal cell carcinoma, sclerosing basal cell carcinoma, morphoeic basal cell carcinoma, fibrosing basal cell carcinoma, BCC, skin cancer

## Abstract

Basal cell carcinoma (BCC) is the most common skin cancer, with a lifetime risk currently approaching up to 40% in Caucasians. Among these, some clinical and pathological BCC variants pose a higher risk due to their more aggressive biological behavior. Morpheaform BCC (morBCC), also known as sclerosing, fibrosing, or morpheic BCC, represents up to 5–10% of all BCC. Overall, morBCC carries a poorer prognosis due to late presentation, local tissue destruction, tumor recurrence, and higher frequency of metastasis. In this systematic review, we review the epidemiological, clinical, morphological, dermatoscopical, and molecular features of morBCC. After the title and abstract screening of 222 studies and the full-text review of 84 studies, a total of 54 studies met the inclusion criteria and were thus included in this review.

## 1. Introduction

Morpheaform basal cell carcinoma (morBCC), also known as sclerosing, fibrosing, or morphoeic basal cell carcinoma, is a histopathologically aggressive subtype of the most common form of skin cancer [1,2]. This subtype is estimated to represent 5–10% of all basal cell carcinomas (BCC), and most commonly arises on the face and neck [3]. Overall, morBCCs carry a poorer prognosis than other BCCs given their higher rates of metastasis, local tissue destruction, and tumor recurrence [2]. Given this, the mainstay of treatment for morBCCs is Mohs micrographic surgery, whereby morBCCs require the most Mohs stages and sections and result in the largest excisional defects given their clinical tumor dimensions [4]. 

Clinically, morBCC commonly presents as a smooth, white- or flesh-colored plaque with areas of induration and ill-defined borders but may also present with erosions or ulcerations within a sclerotic plaque [2]. It is also thought to be the most difficult to diagnose clinically, given that it bears little resemblance to the frequently encountered nodular and superficial BCC. From a histological perspective, morBCC often proves to be a diagnostic challenge, as it may be difficult to distinguish it from other benign adnexal neoplasms, such as trichoepithelioma and desmoplastic trichoepithelioma, syringoma, and microcystic adnexal carcinoma [5,6]. morBCC is characterized by narrow strands and nests of basaloid cells with dense sclerotic stroma, whereby morBCC-involved sections often extend beyond what is observed clinically [7]. To the best of our knowledge, the immunohistochemical and molecular markers or trends specifically associated with morBCC have not been well established.

To this end, a systematic review of the literature was conducted to summarize the clinical and molecular features of morBCC, including demographics as well as morphologic, dermoscopic and histopathologic findings, with the goal of improving the clinical care provided to patients with this high-risk, aggressive form of skin cancer.

## 2. Materials and Methods

### 2.1. Search Strategy

This systematic review’s search was conducted using the PubMed, Embase, MEDLINE, and Scopus electronic databases, using the keywords ‘morpheaform basal cell carcinoma’, ‘morpheaform BCC’, ‘sclerosing basal cell carcinoma’, ‘sclerosing BCC’, ‘fibrosing basal cell carcinoma’, ‘fibrosing BCC’, ‘morphoeic basal cell carcinoma’, and ‘morphoeic BCC’, for articles published from inception to 12 July 2023. Articles were screened independently by author SC using Covidence online systematic review software (www.covidence.org, accessed on 12 July 2023). Eligibility was assessed by scanning the titles and abstracts. All studies reporting clinical presentations and molecular features of morBCC were included. Non-English studies, conference abstracts, nonhuman studies, duplicate presentations, and studies with pathological uncertainty were not included. Full-length articles were then evaluated for mention of clinical or molecular features of morBCC by author SC. The study was not registered in a database such as PROSPERO. 

### 2.2. Data Extraction

SC extracted data independently using a standardized Microsoft Excel form (Microsoft Corporation, Redmond, WA, USA). Variables examined included study design, study size, demographics, morphological features, lesion size, lesion location, dermoscopic features, and positive and negative molecular findings. The quality of evidence was assessed using the Joanna Briggs Institute’s Levels of Evidence.

## 3. Results

**Figure 1:**
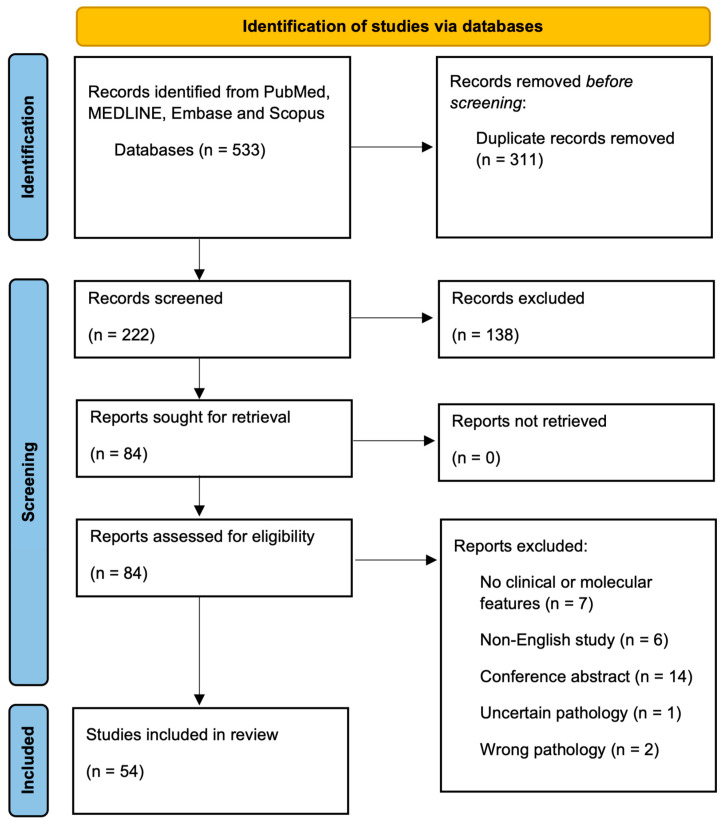
PRISMA Flow Diagram.

After the title and abstract screening of 222 studies and full-text review of 84 studies, a total of 54 studies met inclusion criteria (Figure 1). A total of 23 studies evaluated the clinical features of morBCC (Table 1), while 33 studies focused on the molecular features associated with morBCC (Table 2). Within these, two studies reported both clinical and molecular features. Table 3 provides a short summary of the clinical and molecular findings of our review. 

### 3.1. Clinical Features of morBCC

Twenty-three studies evaluated the clinical features of morBCCs, encompassing 46 patients. Among these, 28 were women and 18 were men, who had a mean age of 62.7 years. Ethnicity was only reported for 13 patients, with breakdown as follows: 7 Caucasian, 2 Japanese, 3 African-American, and 1 Hispanic. Twenty-one articles discussed the morphological features of morBCCs, while four articles reported dermoscopic findings.

With regard to location, the majority of morBCCs noted in the literature were found on the face and head. Breakdown of morBCC location was as follows: nose (n = 10), lip (n = 1), temple (n = 2), eyelid/canthus (n = 3), ear (n = 3), maxilla/cheek (n = 5), eyebrow (n = 1), forehead (n = 2), face (not otherwise specified) (n = 1), scalp (n = 1), chest (n = 2), back (n = 1), and hemiscrotum (n = 1) (Table 1). Sixteen cases reported the size of the examined morBCC, with sizes ranging from 0.5 cm by 0.5 cm to 3.5 cm by 5 cm (Table 1).

A variety of morphological presentations were noted in the literature. Among the most common morBCC-associated clinical features were changes in color (n = 14), ulceration (n = 9 cases) and ill-defined borders (n = 6). Several color changes were noted, with morBCCs being described as hyperpigmented (n = 3), erythematous (redness) (n = 3), white (n = 3), violaceous (n = 1), pearly (n = 1), translucent (n = 1), porcelain-like (n = 1), and yellow (n = 1). Six cases of morBCC were noted to have ill-defined borders compared with only two cases reported as being well defined. morBCCs were described as either mobile or fixed lesions in one case each. Three cases described morBCCs as being firm or hard lesions, while another three studies defined them as being nodular in nature. Moreover, morBCCs were also described as exophytic (n = 2) or morphoeic (n = 1) lesions. One case described morBCC as presenting with a depressed skin surface, while two cases noted raised borders (Table 1). 

Surface changes were also commonly described in the literature. Ulceration (n = 9) was frequently noted, as well as atrophic (n = 5) and sclerotic (n = 4) plaques. Scars or scar-like changes were appreciated in three cases, whereas two cases noted indurated plaques. Other less frequent textural changes included crusting (n = 1), thickness (n = 1), hairlessness (n = 1), scale (n = 1), and a hyperkeratotic surface (n = 1). One case described an morBCC as nonhealing, while another described it as “weeping” (Table 1). 

Miscellaneous features associated with morBCC included bleeding (n = 2), pain (n = 1), and pruritus (itching) (n = 1) (Table 1). 

With regard to dermoscopy, a variety of features were observed. Vascular abnormalities were most reported in the literature, with fifteen cases of arborizing (branched) vessels, five cases of short/superficial telangiectasias, and one report of both linear/serpentine or hairpin vessels. White areas were also frequently noted, with specific features such as white porcelain areas (n = 9), shiny white structures (n = 2), white scale (n = 1), or white clods/milia-like structures (n = 1). Overall, pigmentary changes were a recurrent phenomenon in the morBCCs reported in the literature, with four cases of milky red areas, three cases of black/brown dots, three cases of aggregated yellow-white globules, two cases of blue features (globules or nests), and a single case of a brown-pink background. Moreover, a lack of pigment was appreciated in seventeen cases, and less than 50% extension of pigment was seen in three cases. Structural or textural changes were also noted, with ulceration present in fourteen cases, superficial scale in four cases, follicular criteria in four cases, and chrysalis-like structures and keratin deposition each in a single case (Table 1). 

### 3.2. Molecular Features of morBCC

Thirty-three studies discussed the molecular features of morBCCs (Table 2). A large variety of histopathological and molecular markers were mentioned in the literature. A summary of all the reported markers and their associated function or purpose can be found in Table 4. Among the most reported markers were CK20 negativity (n = 91) and Ki-67 positivity (n = 45), as well as the presence or absence of inflammatory infiltrate (Table 2). 

Cytokeratins, which are cytoskeletal intermediate filament proteins, were commonly discussed in the morBCC literature. The specific identified cytokeratins included CK8 (negative, n = 6), CK15 (positive, n = 20 and negative, n = 9), CK17 (positive, n = 13), CK19 (positive, n = 5; negative, n = 10), CK20 (negative, n = 91), and broad CK (not otherwise specified) (positivity, n = 10). Six cases were found to be positive for CK34-β-E12. 

The literature also delved into a discussion of the molecules known as cluster of differentiation (CD) markers, which are found on the surface of immune cells. More specifically, CD34 was positive in three cases and negative in a single case, and CD23 was negative in five cases. One study commented on the intratumoral or stromal presence of CD4 and CD8, where both intratumoral and stromal CD4 were more common than their CD8 counterparts [49]. 

Several tumor suppressor or cell proliferation markers associated with morBCC were also recognized. Tumor suppressor genes p16 and p53 were positive in five and twenty-nine cases, respectively. Maspin, a product of a tumor suppressor gene, was positive in eight cases. PHLDA1 (or TDAG51) was found to be absent in 31 cases and present in 18 cases. FOXP3, involved in the regulation of the immune system, was negative in 39 cases. In terms of proteins linked to cell differentiation, proliferation, and survival, p63 was positive in 27 cases, Ki-67 in 45 cases, and c-Met in 13 cases. PCNA was found to be positive in seven cases, and proliferation antigens were positive in thirteen cases. Bcl-2 positivity, which prevents cells from undergoing apoptosis, was recorded in 23 cases. GLI1 expression, a transcriptional regulator within the Hedgehog signaling cascade, was found to be significantly reduced in 30 cases. 

Several markers that serve to confirm the epithelial nature of the tissue at hand were also reported. BerEp4 was positive in fifty-four cases, while β-tubulin III was reported in four cases. AE1/AE3, which are commonly detected in epithelial tissues and most carcinomas, were present in 14 cases. COX-2, which stains positively in skin cancers, was found to be more reactive in cases of morBCC than in nodular BCC in 15 cases. 

Markers that may predict more aggressive tumor behavior were also discussed. Fifteen cases of SMA positivity were recorded, while forty cases of αvβ6 positivity were noted. E-cadherin was low in six cases, whose loss is thought to be associated with a gain of tumor cell motility and aggressiveness. Ln-γ2 was positive in 27 cases, and HGF/SF was positive in 13 cases. Ezrin, which is thought to play an active role in regulating tumor growth and progression or dissemination of many cancers, was found to have strong intensity in nine cases. IMP3, on the other hand, was found to be negative in six cases.

Several studies commented on the inflammatory environment of morBCCs. In 15 cases, morBCCs were found to have more inflammation than nodular BCCs at the microscopic level. Fifty-one cases noted a higher mast cell index among morBCCs in comparison with solid BCCs. One study reported a higher presence of lymphoid infiltration (n = 405, 57%) compared with the absence of an inflammatory reaction (n = 306, 43%) among morBCCs [57]. Moreover, 25 cases showed fibroblast-activation protein positivity, contributing to the inflammatory environment. The presence of collagen in morBCCs was also studied throughout the literature, where 10 cases showed an abundance of stromal tissue, mainly collagen, as well as higher type I and III procollagen mRNA levels and steady states compared with normal skin controls. Moreover, collagen VII positivity was noted in four cases. Other physical features common to morBCCs in the literature were the accumulation of microfilaments (n = 4), a lack of hemidesmosomes (n = 4), and an absent or incomplete lamina densa (n = 4).

Neuronal differentiation markers were also reported but were absent across all cases of morBCC. Specific markers included β-tubulin III, GAP-43, and ARC and neurofilament, which were each negative in four cases. Moreover, p75NTR was negative in 12 cases. 

A variety of other markers have been addressed throughout the literature. For ten cases, there was an absence of an epitope identified using a monoclonal antibody that binds the eccrine duct and acrosyringium among morBCCs. Unfortunately, the specifics regarding the epitope were not discussed in the study [30]. Androgen receptor positivity was noted in 40 cases. D2-40, which is used to demonstrate lymphatics and lymphatic differentiation in vascular tumors, was negative in six cases. β-catenin, which plays a role in cell–cell adhesion and whose loss may lead to tumor invasion, was noted to be present in six cases. PD-L1, which is involved in the anticancer immune response, was negative in 39 cases. Four cases were negative for bullous pemphigoid autoantibody.

## 4. Discussion

Given its aggressive nature and high risk for metastasis and recurrence, it is of utmost importance that the clinical features of morBCCs be recognized early and that proper molecular techniques be applied to ensure timely diagnosis and management. 

With regard to clinical features, the majority of the morBCCs included in this review localized to the head and neck, in keeping with the existing literature [73,74,75]. BCCs are thought to be most frequent in this area given their propensity to arise in sun-exposed areas [73]. Only one case, reported by Rohan et al. [28], reported a case of morBCC in a non-sun-exposed area, specifically, the hemiscrotum. However, this cancer arose in a patient known for nevoid basal cell carcinoma syndrome, which is an autosomal-dominant inherited disorder in which patients develop multiple BCCs earlier than the general population due to a variety of mutations in the Sonic hedgehog pathway, namely, in the genes *PTCH1*, *SMO*, *PTCH2*, and *SUFU* [75,76,77]. 

The accepted morphology of morBCC is that of a white- or flesh-colored tumor with areas of induration and ill-defined borders, which may resemble a scar or plaque of morphea [2]. While ill-defined borders were more commonly noted, two separate cases were reported as being well-defined [14,25]. With regard to color, three lesions were noted to be hyperpigmented, while three were noted to be erythematous, differing from the conventional view of morBCCs being white- or flesh-colored. Moreover, while morBCCs are typically regarded as having a smooth surface [2], ulceration was the most frequently reported feature amongst the cases in the literature (n = 9), along with exophytic lesions (n = 2). Overall, it is important to recognize that while some features are more common among morBCC, a high degree of clinical suspicion should be maintained for atypical lesions on the head and neck.

Dermoscopy is an important clinical tool that allows for increased accuracy of BCC detection [78]. Moreover, dermoscopy can provide information on BCC subtype, the presence of pigmentation or ulceration, as well as response rates to a variety of therapies [79]. Although the dermoscopic features of morBCCs were infrequently reported in the literature (n = 4 studies), certain features were repeatedly noted. While telangiectasias and vascular features were frequently reported, these are not unique to morBCC, since vascular patterns in BCCs are considered to be reflective of tumor-associated neoangiogenesis [80]. It is thought that the main dermoscopic features associated with morBCC are pink-white areas and/or fine arborizing vessels and that ulceration is more frequent in this BCC subtype [81], all of which were recognized in this review.

To the best of our knowledge, there is no set grouping of immunohistochemical or molecular markers that should be used to investigate morBCC. In several studies, a variety of markers, namely, fibroblast-activation protein, androgen receptors, Ki-67, p53, Ln-γ2, CK17, p75NTR, and PHLDA1, were used to attempt to differentiate morBCC from other lesions, such as desmoplastic trichoepitheliomas (DTEs), microcystic adnexal carcinomas (MACs), and syringomas, with which morBCCs share clinical similarities [5,31,32,41]. PHLDA-1 negativity, CK17 positivity [44], Ln-γ2 positivity, and p75NTR negativity [35] allow morBCC to be differentiated from DTE, while Ln-γ2 is not useful in the differentiation of morBCC from MAC. However, the presence of BerEp4 can help distinguish between morBCC and MAC [41].

Concerning its pathophysiology, it is thought that the tumor cells of morBCC induce a proliferation of fibroblasts within the dermis and increase collagen deposition [82]. This is consistent with the findings of this review, where higher type I and III procollagen mRNA levels and steady states were found in morBCCs than in normal skin controls [46], as well as positivity of fibroblast-activation proteins among cases of morBCC [43]. At baseline, BCCs are thought to have important inflammatory components, with a connection between tissue destruction, inflammation, and tumor onset [83]. Moreover, morBCCs were found to have more inflammation than nodular BCCs and higher mast cell indices than solid BCCs, reinforcing the notion that morBCCs are more aggressive than their counterparts [45,56]. A need remains to better understand how the tumor microenvironment of morBCC differs from other BCC variants.

Other molecular markers reinforced the notion that morBCCs are more aggressive and confer a high risk for tissue destruction than other BCC subtypes. SMA, which may predict aggressive behavior in cutaneous BCCs, was present in the 15 cases in which it was evaluated [33]. Moreover, integrin ανβ6, which is also associated with a more aggressive phenotype, was positive in all cases in which it was considered [33,34,70]. Other markers whose presence or absence reinforced the notion that morBCCs are aggressive in nature included E-cadherin, Ln-γ2 and HGF/SF [32,55,64]. Conversely, there was a single study in which a molecular marker for aggression, namely, IMP3, was negative in the morBCCs studied [6]. Despite this, there exists substantial evidence in the literature to prove that morBCCs are aggressive in nature, and several of these markers should be applied in clinical practice to improve diagnosis of this form of skin cancer.

We reported a mixed picture with regard to tumor suppressor or cell proliferation markers associated with morBCC in the literature. p16, p53, and Maspin were noted to be positive [6,39,40,42,51]. Interestingly, studies have previously determined that p16, in the context of morBCC/BCC, may also have invasive properties [84,85]. Similarly, Bolshakov et al. found p53 in the majority (66%) of aggressive BCCs [86]; overall, increased p53 expression has been associated with tumor aggressiveness [87], reinforcing the notion that morBCC is an aggressive subtype. Many studies have reviewed the markers of proliferation, such as p63, Ki-67, c-Met, and PCNA, and their association with morBCC [6,7,14,37,42,51]; however, proliferation is unlikely to be unique to morBCC as opposed to BCC, given that previous studies have noted proliferation indices up to 61% among BCCs in general [88]. Despite this, Florescu et al. noted the highest Ki-67 values in the adenoid and morpheaform BCC subtypes [89], suggesting that morBCCs may have higher proliferation rates than other BCC subtypes and thus contribute to their aggressive nature.

This study had several limitations. Firstly, our results are subject to publication bias, as novel or seemingly more interesting findings are published more than their well-defined counterparts. Moreover, only a small number of studies discussing morBCC were present in the literature, limiting the generalizability of results due to the small frequency counts. Finally, given that morBCCs were not always compared with other forms of BCC with regard to molecular characteristics, it is difficult to ascertain whether certain features are specific to morBCCs. 

## 5. Conclusions

Given that morBCCs are highly aggressive and carry a poorer prognosis than their counterparts, both clinical and molecular features must be recognized. Whereas a number of morphological features are more common among morBCCs, physicians should be aware of the plethora of presentations of this form of skin cancer and should be wary of any atypical lesion of the head and neck. Finally, when clinical differentiation of morBCC from other pathologies is difficult, molecular markers should be applied to ensure prompt diagnosis and initiation of the correct management modality, recognizing that morBCCs stain positively for a variety of aggressive molecular markers, tumor suppressor genes, or cell proliferation markers.

## Figures and Tables

**Table 1:** Clinical features of morpheaform basal cell carcinomas from various clinical studies.

	Study Design	Number of Cases	Demographics	Morphology	Location	Dermoscopy
Fayne et al. [8]	Case Report	1	56F, Caucasian	Persistent ulcer	Right nasal ala	None
Itoh et al. [9]	Case Report	1	18F, Japanese	Concave center, edge slightly raised and pigmented, margin indistinct	Upper lip, adjacent to the vermillion border	None
Nadiminti et al. [10]	Case Series	2	Case 1: 80F, African-American	Case 1: Atrophic, sclerotic plaque with peripheral hyperpigmentation and one area of focal nodularity	Case 1: Lateral to the right nasal bridge	None
			Case 2: 61F, African-American	Case 2: Slightly erythematous, ill-defined sclerotic plaque	Case 2: Left lateral nasal tip	
Schanbacher and Randle [11]	Case Report	1	69F, Caucasian	Evidence of scarring, crusting and ulceration Size: 2.5 × 2.1 cm	Right temple	None
Tran et al. [12]	Case Report	1	45M	Ulcerated, weeping, nonhealing Size: 0.5 × 0.5 cm, eventually grew to 1.9 × 2.2 cm	Left lower lateral eyelid without extension past the lateral canthus, which later extended into the inferolateral orbit with bony erosion of the orbital floor	None
Farley et al. [13]	Case Series	2	Case 1: 79M, Hispanic	Case 1: Ill-defined, erythematous, indurated plaque Size: 3.5 × 5.0 cm	Case 1: Right postauricular sulcus and involving the lower third of the auricle, extension of the induration to the earlobe and lower helical rim	None
			Case 2: 57F, Caucasian	Case 2: Painful, bleeding lesion Size: 1.6 × 1.1 cm anterior to and 2.5 × 1.0 cm posterior to the auricle	Case 2: Left preauricular area, which infiltrated the inferior attachment of the auricle to the external auditory canal	
Bains et al. [14]	Case Report	1	26M	Single, well-defined skin-colored atrophic plaque with slightly elevated borders Size: 2.0 × 1.0 cm	Tip of the nose	Brownish-pink background with arborizing vessels, multiple keratin cysts and chrysalis such as structures, black-brown dots, erosion, and whitish scales
Inamura et al. [15]	Case Report	1	82F, Japanese	Ring-form ulceration Size: 3.5 × 1.7 cm	Left temple	Arborizing vessels and ulceration without pigment network
Monroe [16]	Case Report	1	66F	Polygonal, nodular, smooth, scar-like, flat, firm to touch Size: 0.75 cm in diameter	Left maxilla, medial to the nasolabial fold	None
Guttadauro et al. [17]	Case Report	1	58F, Caucasian	Features: Exophytic, irregular, thick, ulcerated, bleeding lesion	Left anterior chest wall	None
Selva et al. [18]	Case Report	1	85F	Indurated plaque with indistinct margins, freely mobile Recurrence post-curettage and electrocautery 11 and 9 years earlier	Right lateral canthus	None
Lucero et al. [19]	Case Report	1	69M	Firm, hairless, violaceous pearly plaque Size: 2.8 × 1.8 cm Recurrence post-excision one year earlier	Left nostril sill, blunting the left philtral column and depressing the superior vermillion lip	None
Costello et al. [20]	Case Series	2	Caucasian men, mean age of 68	None	None	Shiny white structures (2), linear/serpentine vessels (1), milky-red areas (1)
Piva de Freitas et al. [21]	Case Report	1	58M	Ulceroinfiltrative lesion Size: 2.5 × 2.0 cm Recurrence after 3 previous excisions	Extending through the skin of the maxillary region and the left lateral portion of the nose	None
Camela et al. [1]	Case Series	19	7 men, 12 women Mean age of 69.4	None	None	Lack of pigment (n = 16, 84.2%) Arborizing telangiectasias (n = 13, 68.4%) Ulceration (n = 12, 63.2%) White porcelain areas (n = 9, 47.4%) Follicular criteria (n = 2, 10.5%) White clods/milia-like structures (n = 1, 5.3%) Multiple aggregated yellow-white globules (n = 3, 15.8%) Shiny white structures (n = 6, 31.6%) Milky-red structureless areas (n = 3, 15.8%) Superficial scales (n = 4, 21.1%) Keratin mass (n = 3, 15.8%) Short and superficial telangiectasias (n = 5, 26.3%) Hairpin vessels (n = 1, 5.3%) Blue globules (n = 1, 5.3%) Blue nests (n = 1, 5.3%) Brown concentric structures/dots (n = 1, 5.3%) Scattered brown dots (n = 1, 5.3%) < 50% extension of pigment (n = 3, 15.8%)
Rahimizadeh et al. [22]	Case Report	1	28M, Caucasian, HIV+	Ill-defined whitish atrophic plaque Size: 1.9 × 2.9 cm	Left infraorbital and mid cheek areas, in the site of a previous scar	None
Coburn and Scott [23]	Case Report	1	73M	Hard, fixed dermal nodule with normal overlying skin	Outer edge of the left eyebrow	None
Bozikov and Taggart [24]	Case Report	1	61M	Ulceration Size: 3.0 cm in diameter	Left ear lobe, associated with a cigarette burn 2 years prior	None
Nayak et al. [25]	Case Report	1	56F	Hyperkeratotic, hyperpigmented, well-demarcated slightly itchy scaly lesions, some of which were ulcerated, border round and hyperpigmented, center erythematous, scaly, atrophic and ulcerated Size: Ranging from 0.5 × 0.5 cm to 2.0 × 1.5 cm	Chest, forehead, face, scalp and back	None
Gilkes and Borrie [26]	Case Report	1	68F	Ill-defined morphoeic lesion, surface of the skin depressed in some areas with yellowish discoloration	Center of the forehead to the inner canthus of the right eye	None
Litzow et al. [27]	Case Series	3	Case 1: 45F	Case 1: Extensive, translucent, sclerotic plaque that exhibited “classic features” (not further defined) Size: 2.0 × 4.0 cm	Case 1: Left nose and cheek, in the area of a previous scar	None
			Case 2: 43F	Case 2: White sclerotic plaque with the larger portion of the plaque lateral to the patient’s well-healed skin graft Size: 3.0 × 5.0 cm	Case 2: Right cheek and nose	
			Case 3: 49F	Case 3: Whitish lesion Size: 3.0 × 5.0 cm	Case 3: Tip of nose	
Rohan et al. [28]	Case Report	1	58M, known for nevoid basal cell carcinoma syndrome	Exophytic lesion	Left hemiscrotum	None
Lesher et al. [29]	Case Report	1	41F, African-American	Ulcerated, scarred, atrophic, porcelain-colored plaque Size: 1.5 × 2.0 cm	Left side of nose	None

**Table 2:** Molecular features of morpheaform basal cell carcinomas.

	Study Design	Number of Cases	Positive Findings	Negative Findings
Richman and Penneys [30]	Observational Study	10	None	Absence of epitope identified by monoclonal antibody that decorates eccrine duct and acrosyringium in strands of morBCC, whereas eccrine duct stained positively in the same sections
Bains et al. [14]	Case Report	1	Membranous and cytoplasmic expression of BerEp4 Strong nuclear expression of androgen receptor Increased Ki-67 expression in tumor cells with proliferation index of 5–10%	Absence of CD34 staining in the stromal cells between the tumor islands Absence of CK20 positive Merkel cell in the tumor islands
Inamura et al. [15]	Case Report	1	Tumor islands were positive for BerEp4	None
Sellheyer and Krahl [31]	Observational Study	14	None	Tumor keratinocytes were PHLDA1-negative, with the exception of areas of ulceration, whereby PHLDA1 labeled the tumor nests closest to the ulceration and the deeper tumor portions remained nonreactive for PHLDA1
Anderson-Dockter et al. [5]	Observational Study	9	CK17 immunostaining strongly positive in 100% of specimens, detecting individual tumor cells away from tumor strands in 78% (n = 7) of the specimens	none
Koga et al. [32]	Observational Study	28	Ln-γ2 positivity in 96% (n = 27) cases Ln-γ2 expression pattern was different than microcystic adnexal carcinoma (MAC), whereby expression was found in the cytoplasm of tumor cells in morBCC, while in MAC linear expression was noted both along tumor nests and in the cytoplasm BerEp4 positive in 89% (n = 25) cases	CK20-negative in 100% (n = 28) cases
Marsh et al. [33]	Observational Study	13	αvβ6 expression is significantly higher in morBCC compared with nBCC (morBCC = 77%, n = 10, *p* = 0.0009 vs. nBCC = 7%) Strong expression of SMA significantly higher in morBCCs (85%, n = 11, *p* = 0.0036) vs. nBCCs (40%) c-Met strongly or moderately expressed by morBCCs but also in nBCCs HGF/SF commonly detected in myofibroblasts in the desmoplastic stroma	None
Moutasim et al. [34]	Observational Study	30	High expression of αvβ6 (100%, n = 30)	GLI1 nuclear expression significantly reduced (100%, n = 30)
Krahl and Sellheyer [35]	Observational Study	14	None	p75NTR negative in 86% (n = 12) CK20 negative in 100% (n = 14)
Oh et al. [36]	Observational Study	19 Sum of high-risk BCCs: Micronodular, nodular-infiltrative, infiltrative/morpheaform, metatypical	Strong IGF-1R immunoreactivity in 58% of cases (n = 11), with significant difference in IGF-1R expression between low-risk and high-risk BCC (*p* < 0.001) In subgroup analyses, infiltrative/morpheaform BCC (*p* < 0.001) showed significantly different expression of IGF-1R compared with low-risk BCC	None
Vidal et al. [37]	Observational Study	20	p63 positivity in 100% (n = 20); however, all adnexal tumors demonstrated nuclear p63 expression Differences between MAC, DTE, and sBCC were observed with regard to the pattern of staining For morBCC, there was diffuse positivity throughout the tumor mass and without the gradation at different levels of the dermis, as seen in MAC. However, p63 cannot distinguish morBCC from DTE.	None
Carvalho et al. [38]	Observational Study	6	None	CD23-negative in 83% (n = 5)
Bagheri et al. [39]	Observational Study	10	90% of morBCCs (n = 9) showed strong Ezrin intensity, whereby intensity of Ezrin expression was significantly higher in morBCC than in nodular and adenoid types (*p* < 0.001 and *p* = 0.012) No significant difference in expression levels between different types of BCC 80% Maspin positivity (n = 8), but pattern of expression was not different among BCC subtypes	None
Costache et al. [40]	Observational Study	18	Androgen receptors consistently expressed in cases of morBCC Ki-67 positivity in many cells of morBCC in 67% of cases (n = 12) p53 positivity in many cells of morBCC in 78% of cases (n = 14); in few cells, in 22% of cases (n = 4)	Merkel cells were absent in all cases when stained with CK20
Sellheyer et al. [41]	Observational Study	17	BerEp4 positivity in 100% of cases (n = 17); 16 cases revealed immunoreactivity in over 75% and one case in over 25% of cells CK15 reactivity in more than 75% of cells in 35% of cases (n = 6) CK19 positivity in over 75% of tumor cells in 29% of cases (n = 5)	PHLDA1 negativity in 100% of cases (n = 17) CK15 negativity in 53% of cases (n = 9), <25% positivity in an additional 12% of cases (n = 2) CK19 negativity in 59% (n = 10)
Smith et al. [42]	Observational Study	10	Diffuse cytoplasmic staining with AE1/AE3 α-SMA positivity in 40% (n = 4), within the tumor cell, predominantly within peripheral cell populations and was localized to widespread in one case Diffuse Ber-Ep4 expression in 100% of cases (n = 10) Increased CD34 staining of stromal cells surrounding the tumor in 30% of cases (n = 3) Intense p53 expression in more than 25% of the tumor cells in 80% of cases (n = 8) Ki-67 nuclear staining in 20–40% of the cells Bcl-2 expression in 100% (n = 10) cases	Negative for CK7, CK20, EMA, S-100, cerbB2 in 100% of cases (n = 10)
Abbas et al. [43]	Observational Study	25	Fibroblast-activation protein expression was observed in peritumoral fibroblasts in 100% of cases (n = 25) A gradient of fibroblast-activation protein expression was observed, with more intense expression noted in fibroblasts abutting the tumor cells, a less intense expression in the distal peritumoral stromal portion, and minimal to loss of expression in adjacent normal tissue	None
Gamea et al. [44]	Observational Study	4	3 cases (15%) showing grade 3 CK17 expression, 1 case (5%) showing grade 2 CK17 expression CK17 immunostaining clearly detected individual tumor cells away from the dermal tumor strands that seemed nonmalignant with hematoxylin and eosin staining alone	None
Kaiser et al. [45]	Observational Study	15	Higher degree of inflammation (*p* < 0.001) than nodular BCC Higher COX-2 immunoreactivity than nodular BCC (*p* = 0.012), predominantly located on the infiltrating edge of the tumor	None
Moy et al. [46]	Observational Study	10	Electron microscopy demonstrated an abundance of stromal tissue, composed predominantly of collagen fibers. The individual fiber architecture of collagen appeared normal. Elastic fibers with evidence of actinic damage were also present. The connective tissue stroma was often noted in close proximity to tumor cells with prominent nuclei, pronounced rough endoplasmic reticulum and an abundance of mitochondria COL1A1 mRNA level was increased about twofold over that of normal control skin Increase in type III procollagen mRNA in morBCC over normal skin controls Increase in type I and III procollagen mRNA steady-state levels	Type IV procollagen and fibronectin mRNA levels were not different from the controls
Evangelista and North [47]	Observational Study	18	TDAG51 positivity in all cases (n = 18), but all BCC variants stained positive to some degree Slightly higher intensities in TDAG51 staining than CK15 AR was positive in 83% (n = 15) CK15 positive in 78% (n = 14)	CK20 negativity in all cases (n = 18)
Gore et al. [48]	Observational Study	4	GLI1 and GLI2 expressed in the majority of BCCs; however, no specifics for morBCC available	Staining for neurological markers was significantly reduced/negative in morBCC β-tubulin III, GAP-43, ARC, neurofilament all negative in 100% of cases (n = 4) Morphoeic BCCs, which tend to behave more aggressively, stain significantly less than those that behave indolently
Mohanty et al. [6]	Observational Study	6	Mean Ki-67 labeling index was slightly higher for morBCC (8%) than DTE (3%) Beta-catenin expression in all cases (n = 6) p40 expressed in all cases (n = 6) p16 immunoreactivity in 83% of cases (n = 5) ProEx C0positive in 83% of cases (n = 5)	CK20, IMP3, AR, D2-40 negative in 100% of cases (n = 6) No Merkel cells identified in 100% of cases (n = 6)
Gompertz-Mattar et al. [49]	Observational Study	39	Mean of immunohistochemical marker expression: Intratumoral infiltrate 0.19% Intratumoral CD4 27.94% Intratumoral CD8 5.38% Stromal infiltrate 9.78% Stromal CD4 68.78% Stromal CD8 39.29% Stromal FOXP3 0.02% Stromal PD-L1 2.94%	No PD-L1 intratumoral expression in 100% of cases (n = 39) No intratumoral FOXP3 expression in 100% of cases (n = 39)
Quist et al. [50]	Observational Study	Not recorded	CK15, SOX9 and nuclear β-catenin upregulated in comparison with less aggressive tumors Lgr5 positivity in up to 22% of cases of morBCC (*n* not available) Nuclear β-catenin was highly expressed throughout morBCC	Lrig1 and Lgr5 downregulated in comparison with less aggressive tumors
Mateoiu et al. [51]	Observational Study	7	More intense and higher numbers of positive cells for both p53 and the proliferation antigens Peripheral accentuation of both p53 and PCNA in the majority of the tumors PCNA staining was greater than that of Ki-67 Significant Bcl-2 expression	Low Bcl-2 labeling
Erbagci and Erkiliç [52]	Observational Study	34	Mean mast cell index of morBCCs was significantly higher than that of solid BCCs (*p* < 0.02)	None
Klein et al. [53]	Observational Study	44 Sum of all BCC subtypes	Strong and heterogenous reactivity of gp38 in all cases of morBCC (*n* not specified)	None
Jones et al. [54]	Observational Study	4	Collagen VII detection around sclerosing tumor cell populations, which appear to lack bullous pemphigoid (BP) antigen Accumulation of microfilaments along the basal surface where the tumor cells interact with the connective tissue AE3 generates intense staining of both BCC cells and the cells of the epidermis that overlies the patches of tumor cells	BP autoantibody negativity; therefore, lack of BP antigen No obvious hemidesmosomes observed in the tumor cells abutting the connective tissue Lamina densa of the BMZ is either absent or incomplete
Bălăşoiu et al. [55]	Observational Study	6	Low/moderate expression of E-cadherin PCNA proliferation index of more than 60% for the epithelial tumor nuclei 20% expression of Ki-67 of the malignant cells nuclei CK34βE12 presented high expression in the cytoplasm of the tumoral epithelium cells, especially at the core of the tumoral columns High expression of Bcl-2 in 100% of cases (n = 6)	CK8 negativity in 100% of cases (n = 6)
East et al. [7]	Observational Study	10	P63 positivity in 100% of cases (n = 7): intratumoral in 28.5% (n = 2), peripheral in 43% (n = 3), distant in 28.5% (n = 2) Broad-spectrum CK positivity in 100% of cases: intratumoral in 37.5% (n = 3), peripheral in 37.5% (n = 3), distant in 25% (n = 2)	None
Erbagci and Erkilic [56]	Observational Study	17	Mean mast cell index was significantly higher than in other BCC variants (3.46 vs. 2.039, *p* = 0.048)	None
Anthouli-Anagnostopoulou et al. [57]	Observational Study	759	Lymphoid infiltration around tumor nests mild in 20.7% (n = 147), moderate in 25.2% (n = 179), and severe in 11.1% (n = 79)	Absence of inflammatory reaction in 43% (n = 306)

**Table 3:** Short summary of clinical and molecular findings.

Clinical Features	Location	Nose (10)	Eyebrow (1)
		Maxilla/cheek (5)	Scalp (1)
		Eyelid/canthus (3)	Face, not otherwise specified (1)
		Ear (3)	Chest (2)
		Forehead (2)	Back (1)
		Temple (2)	Hemiscrotum (1)
		Lip (1)	
	Morphology	Color changes (14)	Morphoeic (1)
		Ulceration (9)	Mobile (1)
		Ill-defined borders (6)	Fixed (1)
		Atrophy (5)	Depressed skin surface (1)
		Sclerotic plaque (4)	Crusting (1)
		Scar/scar-like change (3)	Thickness (1)
		Firm/hard (3)	Hairlessness (1)
		Nodular (3)	Scale (1)
		Indurated plaque (2)	Hyperkeratotic surface (1)
		Exophytic (2)	Nonhealing lesion (1)
		Well-defined border (2)	Weeping (1)
		Raised border (2)	
	Size	Dimensions between 0.5 cm and 5.0 cm	
	Dermoscopy	Lack of pigment (17)	Blue globules or nests (2)
		Arborizing vessels (15)	Shiny white structures (2)
		Ulceration (14)	White scale (1)
		White porcelain areas (9)	White clods/milia-like structures (1)
		Short/superficial telangiectasias (5)	Brown-pink background (1)
		Milky red areas (4)	Linear/serpentine or hairpin vessels (1)
		Superficial scale (4)	Chrysalis-like structures (1)
		Follicular criteria (4)	Keratin deposition (1)
		Black/brown dots (3)	
		Aggregated yellow-white globules (3)	
		Less than 50% extension of pigment (3)	
Molecular Features	Cytokeratins	CK8 negativity (8)	
		CK15 positivity (20), negativity (9)	
		CK17 positivity (13)	
		CK19 positivity (5), negativity (10)	
		CK20 negativity (91)	
		Broad CK, not otherwise specified positivity (10)	
		CK34-(-E12 positivity (6)	
	Cluster of Differentiation Markers	CD34 positivity (3), negativity (1)	
		CD23 negativity (5)	
		Intratumoral and stromal CD4 more common than CD8	
	Tumor Suppressor Genes and Cell Differentiation and Proliferation Markers	p16 positivity (5)	
		p53 positivity (29)	
		Maspin (8)	
		PHLDA1/TDAG51 positivity (18), negativity (31)	
		FOXP3 negativity (39)	
		p63 positivity (27)	
		Ki-67 positivity (45)	
		c-Met positivity (13)	
		PCNA positivity (7)	
		Proliferation antigens, not otherwise specified positivity (13)	
		Bcl-2 positivity (23)	
		GLI1 decreased expression (30)	
	Epithelial Tissue Markers	BerEp4 positivity (54)	
		(-tubulin III positivity (4)	
		AE1/AE3 positivity (14)	
		COX-2 more reactive in morBCC compared with nodular BCC (15)	
	Markers of Tumor Aggression	SMA positivity (15)	
		αν6 positivity (40)	
		E-cadherin low, not further specified (6)	
		Ln-(2 positivity (27)	
		HGF/SF positivity (13)	
		Ezrin strong intensity (9)	
		IMP3 negativity (6)	
	Markers for Inflammatory Environments	morBCCs more inflammatory than nodular BCCs microscopically (15)	
		Higher mast cell index among morBCCs compared with solid BCCs (51)	
		Higher presence of lymphoid infiltration (405)	
		Absence of an inflammatory reaction (306)	
		Fibroblast-activation protein positivity (25)	
		Abundance of stromal tissue (mainly collagen), higher type I and IIOI procollagen mRNA levels and steady states in morBCCs compared with healthy skin (10)	
		Collagen VII positivity (4)	
		Accumulation of microfilaments (4)	
		Lack of hemidesmosomes (4)	
		Absent or incomplete lamina densa (4)	
	Neuronal Differentiation Markers	β-tubulin III negativity (4)	
		GAP-43 negativity (4)	
		ARC and neurofilament negativity (4)	
		p75NTR negativity (12)	
	Miscellaneous	Absence of epitope binding the eccrine duct and acrosyringium (10)	
		Androgen receptor positivity (40)	
		D2-40 negativity (6)	
		β-catenin positivity (6)	
		PD-L1 negativity (39)	
		Bullous pemphigoid autoantibody (4)	

**Table 4:** Markers positively or negatively associated with morBCC, with corresponding dermatopathological purpose. Information taken from https://www.pathologyoutlines.com/ unless otherwise specified (accessed on 11 August 2023).

Marker	Function
AE1/AE3	Immunoreactivity is observed in epithelia and most carcinomas
AR	Transcription factor facilitating the effects of androgens, expressed variably across breast cancer subtypes
ARC	Neuronal differentiation marker [48]
Bcl-2	Prevents cells from undergoing apoptosis
BerEp4	Membranous staining; antibody to cell membrane glycoproteins expressed on healthy epithelia and in various carcinomas
c-Met	Activates various signaling pathways that lead to proliferation and cell survival
CD23	To differentiate small lymphocytic lymphoma/chronic lymphocytic leukemia from mantle cell lymphoma or MALT lymphoma; B cell marker
CD34	Distinguish Kaposi sarcoma, dermatofibrosarcoma protuberans, and epithelioid sarcoma from dermatofibroma; distinguish solitary fibrous tumor from desmoplastic mesothelioma; distinguish hemangiopericytoma from endometrial stromal sarcoma
cerbB2	Crucial role in cell growth and division, specifically associated with HER2+ breast cancer
CK15	Downregulated in activated keratinocytes in psoriasis, hypertrophic scars, and skin injury; normal positive staining in nail, hair follicle bulge, and follicular stem cells; positive staining in trichoepithelioma
CK17	Basal type cytokeratin of complex epithelia; positive staining in basal cell carcinomas, hair shaft epithelia, and sebaceous glands
CK19	Present in simple and complex epithelium; positive staining in hair follicles; negative stain in trichilemmoma
CK20	Epithelial marker; positive staining in Merkel cell carcinoma and fibroepithelioma of Pinkus
CK34βE12	Positive staining in classic and basaloid squamous cell carcinoma, as well as amyloid deposits associated with squamous cell carcinoma and dysplasia in the head and neck
CK8	Used to confirm epithelial nature of tissue/tumors
COL1A1	Major component of type I collagen, overexpressed in many cancers across numerous cellular processes [58]
COX-2	Positive staining in skin cancers
D2-40	Mostly used to show lymphatics (e.g., lymphovascular invasion) and lymphatic differentiation in vascular tumors; positive in primary skin adnexal tumors
E-cadherin	Transmembrane protein involved in cellular adhesion and polarity maintenance; loss is associated with gain of tumor cell motility and invasiveness
EMA	Absent in normal epithelia, but highly positive staining in most carcinomas
Ezrin	Links the cell membrane and the actin cytoskeleton; cell adhesion to the extracellular matrix, cell–cell communication, signal transduction, and apoptosis; active role in regulating tumor growth and progression and metastatic dissemination of many cancers [39]
FOXP3	Plays an essential role in maintaining homeostasis of the immune system by regulating the suppressive function, stability, and expansion of Tregs; facilitates tumorigenesis by enabling tumor cells to evade antitumor immunity by inhibiting T-cell proliferation
GAP-43	Neuronal differentiation marker [48]; display intrinsic oncogenic functions [59]
GLI1	Important transcriptional regulator within the Hh signaling cascade [60]; specifically expressed in the bulb areas of hair follicles [61]
GLI2	Essential for embryonic hair follicle development [62]
gp38	Mucin-type protein upregulated in several squamous cell carcinomas, along with their corresponding CAFs [63]
HGF/SF	Expressed by myofibroblasts [33]; stimulates motility and invasiveness of epithelial and cancer cells [64]
IGF-1R	Highly overexpressed in various carcinomas, promoting cell survival through its function as an antiapoptotic agent [65]
IMP3	Cytoplasmic marker with expression in many malignancies; tendency of higher expression in more aggressive neoplasms
Ki-67	Marker of cell proliferation; increased in most malignant and inflammatory conditions
Lgr5	Hair follicle stem cell marker [50]
Ln-γ2	Marker of invasive tumors; frequently expressed in malignant tumors [32]
Lrig1	Positive prognostic marker in Merkel cell carcinoma [66]
Maspin	Product of a tumor suppressor gene; involved in apoptosis and inhibition of carcinoma invasion, metastasis, and angiogenesis; expression is downregulated during cancer progression [39]
p16	Tumor suppressor protein
p40	Stimulates cell proliferation, blocks apoptosis, and favors unrestrained tumor growth
p53	Tumor suppressor gene
p63	Regulates human keratinocyte proliferation; myoepithelial marker; does not appear to be a tumor suppressor gene
P75NTR	Controlling the survival and process formation of neurons [67]
PCNA	Role in DNA synthesis, DNA repair, and cell cycle progression; expression correlates with proliferation activity
PD-L1	Immune checkpoint protein expressed on activated immune cells and tumor cells; coinhibitory factor to regulate the immune response and limit autoimmunity; adaptive resistance mechanism to avoid T cell mediated anticancer immune response
PHLDA1/TDAG51	Tumor suppression; to differentiate trichoepithelioma from BCC
ProEx C	Helpful in distinguishing melanoma from benign nevi; useful proliferation marker for high-grade vulvar or cervical intraepithelial neoplasia [68,69]
S-100	Tumor marker of metastatic melanoma, along with clear cell sarcoma of soft tissue and myoepithelial tumors
SMA	Identifies pericytes, myoepithelial cells, smooth muscle cells and myofibroblasts in normal, reactive, or neoplastic tissue; immunoexpression may predict aggressive behavior in cutaneous basal cell carcinoma
SOX-9	Transcription factor linked to hedgehog pathways, plays a central role in development and differentiation of multiple cell lineages
αvβ6	Regulate epithelial remodeling during development, tissue repair, and neoplasia; associated with a more aggressive phenotype [70]
β-catenin	Mutations and overexpression of β catenin are associated with various carcinomas; plays an important role in the cadherin/catenin complex dynamics involved in cell–cell adhesion, the loss of which may lead to tumor invasion and metastasis [71]
β-tubulin III	Neuronal differentiation marker [48]; frequently overexpressed in human tumors and associated with tumor aggressiveness [72]

## Data Availability

No new data were created or analyzed in this study. Data sharing is not applicable to this article.
